# Dissecting complex transcriptional responses using pathway-level scores based on prior information

**DOI:** 10.1186/1471-2105-8-S6-S6

**Published:** 2007-09-27

**Authors:** Harmen J Bussemaker, Lucas D Ward, Andre Boorsma

**Affiliations:** 1 Department of Biological Sciences, Columbia University, 1212 Amsterdam Avenue, MC 2441, New York, NY 10027, USA; 2 Center for Computational Biology and Bioinformatics, Columbia University, New York, NY, USA; 3 Swammerdam Institute for Life Sciences, University of Amsterdam, BioCentrum Amsterdam, Nieuwe Achtergracht 166, 1018 WV Amsterdam, The Netherlands

## Abstract

**Background:**

The genomewide pattern of changes in mRNA expression measured using DNA microarrays is typically a complex superposition of the response of multiple regulatory pathways to changes in the environment of the cells. The use of prior information, either about the function of the protein encoded by each gene, or about the physical interactions between regulatory factors and the sequences controlling its expression, has emerged as a powerful approach for dissecting complex transcriptional responses.

**Results:**

We review two different approaches for combining the noisy expression levels of multiple individual genes into robust pathway-level differential expression scores. The first is based on a comparison between the distribution of expression levels of genes within a predefined gene set and those of all other genes in the genome. The second starts from an estimate of the strength of genomewide regulatory network connectivities based on sequence information or direct measurements of protein-DNA interactions, and uses regression analysis to estimate the activity of gene regulatory pathways. The statistical methods used are explained in detail.

**Conclusion:**

By avoiding the thresholding of individual genes, pathway-level analysis of differential expression based on prior information can be considerably more sensitive to subtle changes in gene expression than gene-level analysis. The methods are technically straightforward and yield results that are easily interpretable, both biologically and statistically.

## Introduction

Many of the popular methods for analyzing DNA microarray expression data, from clustering [1] to more sophisticated machine-learning approaches [2-5], require expression data over a large number of different conditions as input. However, it is common to only have expression data for a few different strains and/or conditions. In this case, what is of interest are the changes in mRNA abundance for each gene, usually represented as the logarithm of the fold-change between test and control. The traditional way of analyzing such data is to first identify significantly up- and down-regulated genes, and subsequently to characterize these sets in terms of enrichment for functional annotation [6] or upstream promoter elements [7-9]. However, by requiring statistically significant differential expression at the level of individual genes, a lot of information about differential expression will be lost that could have been detected using analysis methods working at the level of pathways.

To understand this, assume that we are comparing two conditions and that the measurement error for the fold-change of individual genes is 20%. Now consider a specific pathway consisting of 100 genes that are all upregulated by 10%. This level of differential expression is well within the noise for individual genes, none of which will therefore be classified as significantly induced. However, the error in the *average *expression of 100 *randomly *chosen genes will be on the order of 20%/$\sqrt{100}$ = 2%. The 10% change in expression at the level of the whole pathway therefore corresponds to five units of standard error and is highly statistically significant.

**Figure 1:**
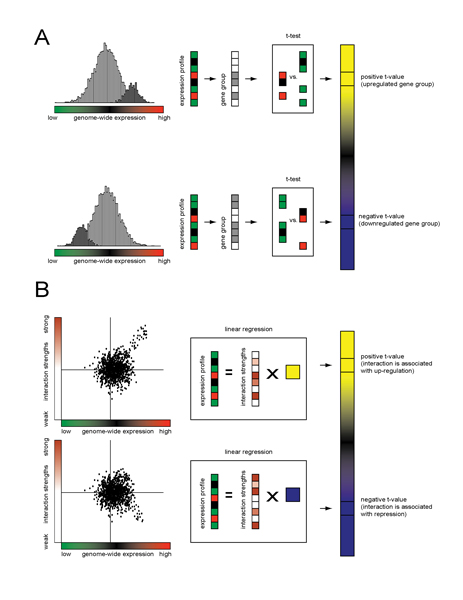
**Scoring pathway activity: gene sets versus regression**. Two types of prior information, categorical and quantitative, may be combined with non-thresholded genome-wide expression data to derive a statistical measure of pathway-level activity. In (A) a pre-defined gene set (gray), such as those annotated by the Gene Ontology project, is scored using a t-test for its expression response (red = positive, green = negative) compared to all other genes. In (B) estimated interaction strengths (shades of gray), such as those derived from regulatory sequence analysis or ChIP-chip experiments, are correlated with the expression response of all genes. In both instances the result is a t-value (yellow = positive, blue = negative) that measures the change in mRNA expression associated with a category (A) or interaction (B).

In recent years, two distinct classes of methods have been developed that use prior information about how genes can be viewed as belonging to different regulatory or functional pathways (Figure 1). This information can be used to score differential expression at the pathway level rather than at the gene level. The first class of methods represents pathways as gene sets, to which individual genes either belong or do not belong. One well-known source of such gene sets is the Gene Ontology (GO) project [6], where the classification is based on the function of the proteins encoded by each gene. The second class of methods takes a more sophisticated approach by assigning a regulatory susceptibility to each gene, quantifying how strongly this gene is expected to respond to a change in the activity of a specific regulatory pathway. For example, the affinity of a gene's promoter sequence for a specific transcription factor (TF) could be predicted using consensus motifs or weight matrices [10] and be used to predict the response of that gene to changes in TF activity.

In this review, we describe how such pathway-level analyses can be implemented mathematically. It is helpful to understand that, in general, information about genes comes in two different types: *categorical *information of boolean type ("true" or "false"), which tells us whether or not a gene belongs to a specific gene set; and *quantitative *information, e.g., the mRNA expression log-ratio between two conditions for a gene or the ChIP-chip [11] fold enrichment for the gene's promoter region. Given any two distinct features characterizing each gene, their genomewide statistical association can be scored using an appropriate statistical test (Table 1).

**Table 1:** When to use which statistical test.

First Feature	Second Feature	(Non)-Parametric?	APPROPRIATE TEST
categorical	categorical	non-parametric	hypergeometric
quantitative	quantitative	parametric	Pearson
quantitative	quantitative	non-parametric	Spearman, Kendall
categorical	quantitative	parametric	two-sample t-test
categorical	quantitative	non-parametric	Wilcoxon-Mann-Whitney, Kolmogorov-Smirnov

When analyzing the statistical association between two features across the genome, the choice of statistical test depends on whether the features are categorical or quantitative, and whether or not a parametric method can be used. For each case the appropriate test is listed.

### The traditional approach: scoring over-representation of predefined gene sets

Suppose that we want to know whether a specific set of genes of interest is statistically enriched for genes with a specific annotation in Gene Ontology. In this case, both features (namely, "does the gene belong to the set of genes of interest" and "is the gene associated with GO term X") are categorical, and the appropriate statistic is the *overlap *between both gene sets. Let the total number of genes in set *A *be *a*, the total number of genes in set *B *be *b*, and the total number of genes in the genome be *n*. Furthermore, let the overlap *x *denote the number of genes shared between *A *and *B*. If the two sets are chosen randomly and independently, the average overlap will be:

(1) $$\langle x\rangle =\frac{ab}{n}$$

This makes sense: if a fraction *b/n *of all genes belongs to set *B *then the expected fraction of genes in set *A *that also belongs to set *B *equals *x/a*. In the case of over-representation, when *x *> <*x*>, the P-value that quantifies how likely it is to get at least the same number of overlapping genes by chance, is given by

(2) $$P_{\text{over}}(x)=\sum_{x^{\prime }=x}^{\min(a,b)}H(x^{\prime }|a,b,n)$$

where *H *is the hypergeometric distribution given by

(3) $$H(x|a,b,n)=\frac{\left(\begin{array}{c} a\\ x \end{array}\right)\left(\begin{array}{c} n-a\\ b-x \end{array}\right)}{\left(\begin{array}{c} n\\ b \end{array}\right)}$$

and

(4) $$\left(\begin{array}{c} n\\ k \end{array}\right)=\frac{n!}{k!(n-k)!}$$

It is also possible to have significant under-representation (*x *< <*x*>). In that case, the P-value is given by

(5) $$P_{\text{under}}(x)=\sum_{x^{\prime }=0}^{x}H(x^{\prime }|a,b,n)$$

This use of the cumulative hypergeometric distribution is also known as "Fisher's exact test." The test is by nature non-parametric because both input features are non-parametric. Under specific conditions the hypergeometric distribution may be approximated by the binomial or chi-square distribution. Several implementations of this approach are reviewed by Khatri and Draghici [12]. Since typically a large number of gene sets are scored in parallel, the p-values must be corrected for multiple testing. Grossman et al. [13] recently addressed technical complications arising from the strong overlap between the hierarchically organized Gene Ontology categories.

### An alternative: scoring the distribution of expression levels for predefined gene sets

An early example of the use of predefined gene sets to analyze differential expression at the pathway level can be found in Lascaris et al. [14]. The authors used a z-score to represent the difference between the average expression in a gene set *S *consisting of *n *genes and the genomewide mean *μ*:

(6) $$z=\frac{{\bar{x}}_{S}-\mu }{\sigma_{\bar{x}}}$$

Here $\sigma_{\bar{x}}$ = *σ*/$\sqrt{n}$ is the standard error of the mean, *σ *being the standard deviation of the genomewide distribution of log-ratios. The same metric is used by the "parametric analysis of gene expression" (PAGE) method of Kim and Volsky [15]. For larger gene sets, however, the standard t-test for the difference between means yields more accurate results [16]. The t-test, in general, scores the statistical association between a categorical and quantitative feature. The categorical feature is used to partition the set of all genes, *G*, into two complementary subsets *S *and S'. $\bar{S}$The *t *statistic measures the difference between the means of the two subsets in units of its standard error:

$t=\frac{{\bar{x}}_{S}-{\bar{x}}_{S^{\prime }}}{\sigma \sqrt{\frac{1}{\left|S\right|}+\frac{1}{\left|S^{\prime }\right|}}}$

Here $\bar{x}$_*S *_and ${\bar{x}}_{S^{\prime }}$ are the mean expression value of genes in set *S *and *S'*, respe$\bar{x}$ctively, and the standard error of the difference is given by

(8) $$s=\sqrt{\frac{(|S|-1)\sigma_{S}^{2}+(|S^{\prime }|-1)\sigma_{S^{\prime }}^{2}}{|S|+|S^{\prime }|-2}}$$

with *σ*_*S *_and *σ*_*S' *_the standard deviation of the expression values of the genes within set *S *and *S'*, respectively. Using a t-distribution with *n *- 2 degrees of freedom, each t-value can be converted to a p-value, which should again be corrected for multiple testing.

**Figure 2:**
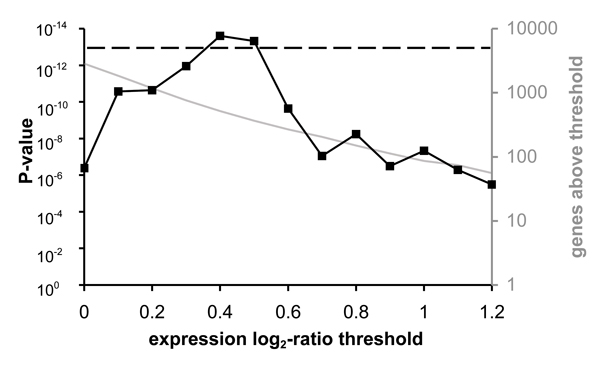
**Scoring GO categories: Fisher's exact test versus two-sample t-test**. We analyzed gene expression data for the response to the ergosterol biosynthesis inhibitor Lovastatin as measured by Hughes et al. [27]. The two-sample t-test reveals that the mean expression level of genes in the GO category "ergosterol biosynthesis" is significantly higher than expected (dotted line; *t *= 7.4; *P *= 1.1·10^-13^). Fisher's exact test can be used to score over-representation of the same GO category in the set of most induced genes. However, this requires one to first define a threshold for the expression fold-change of individual genes. The solid line shows how the P-value from Fisher's exact test depends on this threshold.

Figure 2 shows a side-by-side comparison of Fisher's exact test and the t-test for a specific combination of GO category and genomewide differential expression profile. Fisher's exact test can only be applied once a set of "genes of interest" has been defined. We thresholded the fold-induction of individual genes to define this gene set, and computed GO category enrichment P-values at different thresholds (solid line/symbols). The smallest, most significant, P-value is obtained at an individual-gene threshold significantly below 2-fold induction, satisfied by over 500 genes. In general, the optimal threshold will depend on both the GO category and the expression data. By contrast, the two-sample t-test uses the expression value for all genes; no threshold for individual genes is required, an important practical advantage. While the optimal P-value from Fisher's exact test is slightly smaller than that of the two-sample t-test (dashed line), this seeming advantage disappears as soon as multiple-testing correction associated with the required threshold optimization is taken into account. Note that at the commonly used threshold of 2-fold induction, the two-sample t-test performs dramatically better.

Other statistical tests have also been used to detect differential expression of gene sets based on the distribution of expression values. The original version of the "gene set enrichment analysis" (GSEA) method [17] used the Kolmogorov-Smirnov (KS) statistic to test whether the distribution of expression levels in a specific gene set was different from that of all genes; this approach was later found to require a modification to work reliably [18]. The Wilcoxon-Mann-Whitney test, a non-parametric equivalent of the t-test that uses expression values only to rank the genes, has also been applied to this problem [19].

### Beyond gene sets: approaches based on regression analysis

The assignment of genes to gene sets is categorical: Either the gene belongs to the set, or it does not. However, gene sets are often a proxy for regulatory pathways. This is most obvious in the case of the gene sets based on ChIP-chip data [11], which were used by Boorsma et al. [16] to analyze differential mRNA expression using the t-test. The strict delineation of "targets" of a given TF based on thresholding of the ChIP-chip signals is an oversimplification. In reality, the degree to which the transcription rate for a given gene responds to a change in the activity of the TF depends in a continuous fashion on the binding affinity between the TF and the promoter DNA (as well as interactions with co-factors, chromatin, etc.). Thus, if an estimate of this affinity is used as a predictor for changes in transcription rate (and therefore expression), a single parameter that quantifies the global change in TF activity may explain a wide range of transcriptional responses across the genome. This intuition can be formalized in the form of a linear regression model:

(9) *A*_*g *_= *C *+
*FN*_*g*_

where *C *is an intercept and *F *a slope estimating the change in TF activity. The dependent ("response") variable *A*_*g *_is the mRNA expression log-ratio of gene *g *between conditions. The independent ("predictor") variable *N*_*g *_represents the regulatory network connectivity between the TF and the promoter region of gene *g*. For given *A*_*g *_and *N*_*g*_, the deviance *D *between the measured and predicted expression values

(10) $$D=\sum_{g}{\left(A_{g}-C-FN_{g}\right)}^{2}$$

is minimized. The solution is given by

(11) $$F=\frac{\langle AN\rangle -\langle A\rangle \langle N\rangle }{\langle N^{2}\rangle -{\langle N\rangle }^{2}}=\frac{\langle \delta A\delta N\rangle }{\langle \delta N^{2}\rangle }$$

and

(12) *C *= <*A*> - *F
*<*N*>.

where <*X*> = (1/*G*) ∑_*g *_*X*_*g *_denotes an average over all genes and *δX*_*g *_≡ *X*_*g *_- <*X*> denotes the deviation from the genomic mean, so that <*δX*^2^> equals the variance of *X*. Because we are dealing with *univariate *regression (a single independent variable), the Pearson correlation coefficient between *A *and *N*,

(13) $$r=\frac{\langle \delta A\delta N\rangle }{\sqrt{\langle \delta A^{2}\rangle \langle \delta N^{2}\rangle }}$$

can be directly related to the slope *F *by the following equation:

(14) $$F=r\sqrt{\frac{\langle \delta A^{2}\rangle }{\langle \delta N^{2}\rangle }}$$

It can furthermore be shown that, in the univariate case, *R*^2^, defined as the fraction of the variance in expression that can be explained by the linear model, is given by the square of Pearson correlation:

(15) $$R^{2}=\frac{\mathrm{var}(C+FN)}{\mathrm{var}(A)}=\frac{{\langle \delta A\delta N\rangle }^{2}}{\langle \delta A^{2}\rangle \langle \delta N^{2}\rangle }=r^{2}$$

A transformation of *r *due to R.A. Fisher

(20) $$t=\frac{1}{2}\ln\left(\frac{1+r}{1-r}\right)$$

yields a statistic *t *that is distributed according a t-distribution with *n *- 3 degrees of freedom, and can thus be easily converted to a p-value. Again, multiple testing will need to be accounted for whenever the association with multiple features is scored in parallel.

There are many ways in which the regulatory network connectivities *N*_*g *_can be chosen. The first application of regression analysis to microarray data, by Bussemaker et al. [20], used integer motif counts in promoter regions. Continuous sequence scores based on position-specific scoring matrices (PSSMs) [21,22] and position-specific affinity matrices (PSAMs) [23,24] have also been used. The values for *R*^2 ^obtained with such sequence-based predictors are typically in the range of 1–5%. Another possible choice for *N *are ChIP-chip enrichment (log-)ratios [25,26]. As these values are relatively noisy experimental measurements, the values for *R*^2 ^observed in this case are usually smaller (< 1%).

## Conclusion

In this work, rather than providing a comprehensive review of all relevant literature, we have outlined two conceptually different approaches for scoring differential expression at the pathway level. These methods use prior information about how different genes relate to each other to reduce the dimensionality of the problem. This obviates the need to first obtain gene clusters or modules from expression data over multiple conditions, and thereby makes it possible to analyze each differential expression profile by itself in a condition-specific fashion.

## Authors' contributions

HJB drafted the paper, which was edited and proofread by all authors. LDW and AB prepared Figure 1 and 2, respectively.
